# Transcatheter Periarticular Embolization via Brachial Artery Access in Elderly Patients With Primary Hip Osteoarthritis: A Pilot Prospective Analysis

**DOI:** 10.7759/cureus.96060

**Published:** 2025-11-04

**Authors:** Andrei Marian Feier, Florin A Bloj, Octav Marius Russu, Andrei Bloj, Sandor Gyorgy Zuh, Tudor Sorin Pop

**Affiliations:** 1 Department M4 Clinical Sciences, Orthopedics and Traumatology I, George Emil Palade University of Medicine, Pharmacy, Science, and Technology of Targu Mures, Targu Mures, ROU; 2 Ares Excellence Center, Memorial Baneasa Hospital, Bucharest, ROU; 3 Department 8, Radiology, Medical Imaging, and Interventional Radiology II, Carol Davila University of Medicine and Pharmacy, Bucharest, ROU

**Keywords:** hip osteoarthritis, interventional radiology-guided embolization, lateral circumflex femoral artery, minimally invasive, transarterial embolization

## Abstract

Background

Hip osteoarthritis (HOA) is increasingly prevalent and imposes substantial economic and quality-of-life burdens. Vascular dysregulation is a modifiable pain driver. Transarterial embolization (TAE) of joint vessels is established for knee osteoarthritis, but prospective data for the hip are nonexistent. The main aim of this study was to evaluate mid-term pain relief and functional improvement after TAE in elderly patients with mild-to-advanced HOA who were ineligible for total hip arthroplasty. Secondary endpoints included assessing overall procedural safety and technical feasibility.

Methodology

In this prospective, single-arm study, a sample size of 18 consecutive patients (mean age ± SD = 67.2 ± 5.7 years; 11 women) with Tönnis grade I-II HOA, ≥1 surgical contraindication, and symptoms for >1 year underwent TAE via brachial access using Nexsphere-F™ bioresorbable microspheres (100-300 μm). Endpoints were changes in Visual Analog Scale (VAS) pain and Harris Hip Score (HHS) at one, four, eight, and twelve weeks versus baseline.

Results

VAS decreased from 7.8 ± 1.3 at baseline to 4.2 ± 2.0 at week one and 4.3 ± 2.2 at week twelve (Δ -3.5 points at 12 weeks; p < 0.01). HHS improved from 45.5 ± 4.7 at baseline to 59.9 ± 7.1 at week one and 62.1 ± 7.5 at week twelve (Δ +16.6 points; p < 0.01). No adverse events occurred.

Conclusions

Hip TAE produced rapid and durable reductions in pain and improvements in hip function without adverse events in elderly, surgery-ineligible HOA patients using brachial access and bioresorbable microspheres. These findings support TAE as a promising minimally invasive bridge between conservative therapy and arthroplasty.

## Introduction

Hip osteoarthritis (HOA) has become more common during the past three decades. The age-standardized incidence increased from 17.02 to 18.70 per 100,000 persons between 1990 and 2019, and radiographic changes are present in an estimated 5-25% of adults older than 55 years [1,2]. The disorder also has a sizeable macroeconomic drag, with its direct and indirect costs absorbing roughly 1-2.5% of gross national product in high-income economies [3]. Lost productivity is a major driver: in the Netherlands, a typical HOA-related sick leave lasts several months and results in substantial individual costs, with national absenteeism losses reaching millions of euros annually [4]. Health-related quality of life is also reduced, with recent assessments showing wide variation depending on disease severity [5].

Although mechanical overload remains a main cause of wear and tear, mounting basic science and imaging evidence points to vascular contribution to disease progression [6]. Episodic reductions in subchondral perfusion, venous congestion, and microembolization induce intraosseous hypertension, hypoxia, and osteocyte apoptosis, accelerating cartilage loss [7]. Dynamic-contrast MRI, contrast-enhanced ultrasound, and angiography frequently demonstrate patchy periarticular arterial branches and venous hypervascularization, while upregulated vascular endothelial growth factor drives synovial and subchondral neovascularization; nascent vessels, accompanied by sensory nerve branches, breach the normally avascular osteochondral junction and amplify nociceptive signaling [8-11]. Together, these observations suggest that selectively reducing hypervascularity relieves pain and slows structural deterioration. Total hip arthroplasty (THA) remains the gold-standard treatment for advanced HOA, yet a substantial proportion, estimated at 15-30% of patients, are considered unsuitable for THA due to comorbidities such as active infection, severe cardiovascular disease, morbid obesity, poor glycemic control, or high anesthetic risk [9,12,13]. For this subgroup, current non-operative options (analgesics, intra-articular injections, physiotherapy) provide only transient relief. Transarterial embolization (TAE) has emerged as a minimally invasive technique that targets pathological periarticular hypervascularity and has shown encouraging results in knee osteoarthritis [14]. TAE for osteoarthritis consists of superselective catheterization of periarticular branches that supply the synovium and subchondral plexus, angiographic identification of neovascular “blush,” and controlled TAE with calibrated microspheres to achieve stasis [14]. The therapeutic goal is to devascularize aberrant neovessels and their accompanying nociceptive fibers to reduce pain and inflammatory drive.

Hip data, however, are confined to short case reports and small series; no prospective cohort has yet examined patients with mild-to-advanced disease who have exhausted conservative care and are unsuitable for THA [15]. Given the evolving field of minimally invasive options for osteoarthritis, a comprehensive evaluation of hip TAE is mandatory. While intra-articular injections, platelet-rich plasma, radiofrequency ablation, and nerve blocks are increasingly used, none specifically target the vascular mechanisms now recognized as contributors to pain and progression [16]. By addressing periarticular hypervascularity, TAE represents a mechanistically distinct strategy that complements or precedes these modalities. Expanding the evidence base for hip TAE is therefore critical to defining its role in treatment algorithms. This study aims to assess the outcomes of TAE in terms of pain relief and functional improvement after hip TAE in elderly patients with advanced HOA and at least one absolute or relative contraindication to THA.

## Materials and methods

Study design and patient selection

This was a prospective, single-arm trial designed to evaluate the clinical efficacy of TAE in elderly patients diagnosed with mild and advanced primary (idiopathic) HOA and contraindication to THA. Eligible patients were recruited based on predefined inclusion criteria, which included age (60 years or older), radiographic evidence of HOA (Tönnis grade I and II), minimum of two prior conservative management therapies according to the Osteoarthritis Research Society International (OARSI) [3], duration of symptoms >1 year, minimum of one contraindication to surgical treatment (uncontrolled diabetes, severe cardiovascular disease, chronic obstructive pulmonary disease, body mass index ≥40 kg/m², HbA1c >8%, heavy smoking). Group structure based on Tönnis grade is represented in Table 1.

**Table 1:** Patients screened and selected based on Tönnis grade and THA contraindications. THA = total hip arthroplasty; BMI = body mass index

Category	Stage/Type	Number (percentage)
Screened	-	113
Selected	-	18 (16%)
Tönnis grade	I	7 (39%)
	II	11 (61%)
THA contraindications	Severe cardiac disease	8 (44%)
	High BMI	9 (50%)
	Heavy smoker	3 (17%)

**Figure 1:**
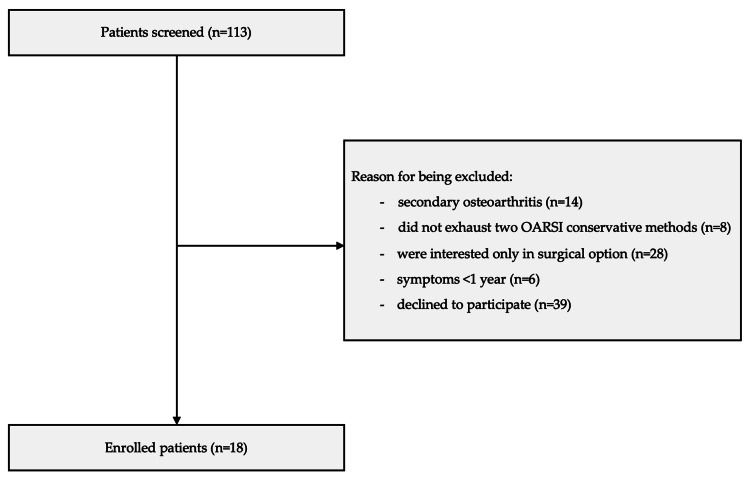
Flow diagram showing screening, exclusions, and enrolled participants. OARSI = Osteoarthritis Research Society International

Before enrollment, all patients provided informed consent after a thorough explanation of the study objectives, benefits, and risks associated with the intervention. This was a pilot study, and the sample size of 18 patients was determined based on expected recruitment over a six-month period and institutional capacity. Participant screening is presented in the flowchart illustrated in Figure 1.

Outcomes and assessments

Outcome assessments were performed using the Visual Analog Scale (VAS) and Harris Hip Score (HHS). These assessments were administered preprocedural, at one week, and at four, eight, and twelve weeks after TAE. Secondary endpoints included the assessment of procedural safety and technical success.

Interventional procedure

**Figure 2:**
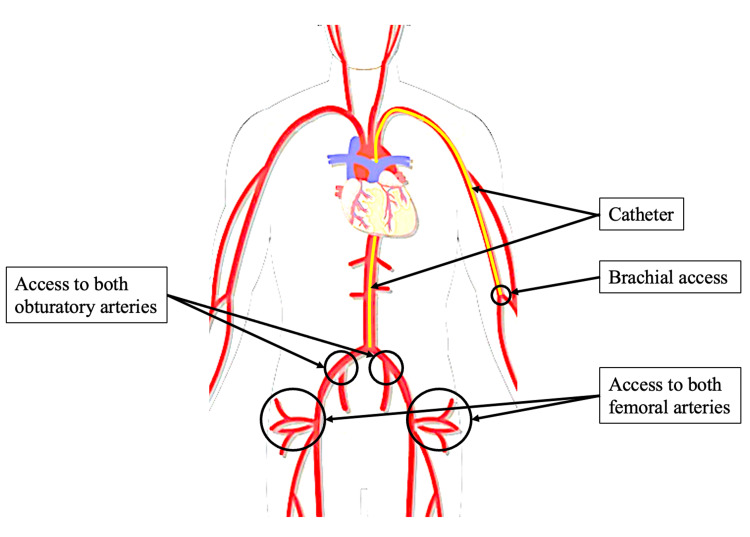
Overview of left brachial arterial access and catheter trajectory to the femoral and obturator arteries targeted during hip transarterial embolization. The curved yellow line indicates typical microcatheter course from the left brachial access point.

A mixture containing contrast and Nexsphere-F™ is used as an embolization agent as follows: one vial of Nexsphere-F™ diluted in 5 mL of saline + Iodixanol (Visipaque®, GE Healthcare Limited, Chalfont St. Giles, United Kingdom). The procedure begins with catheterization of the left brachial artery, followed by the advancement of a 4 Fr, 125 cm multi-purpose catheter (MP) to the level of the internal iliac artery (IA) under fluoroscopic guidance (Figure 2).

**Figure 3:**
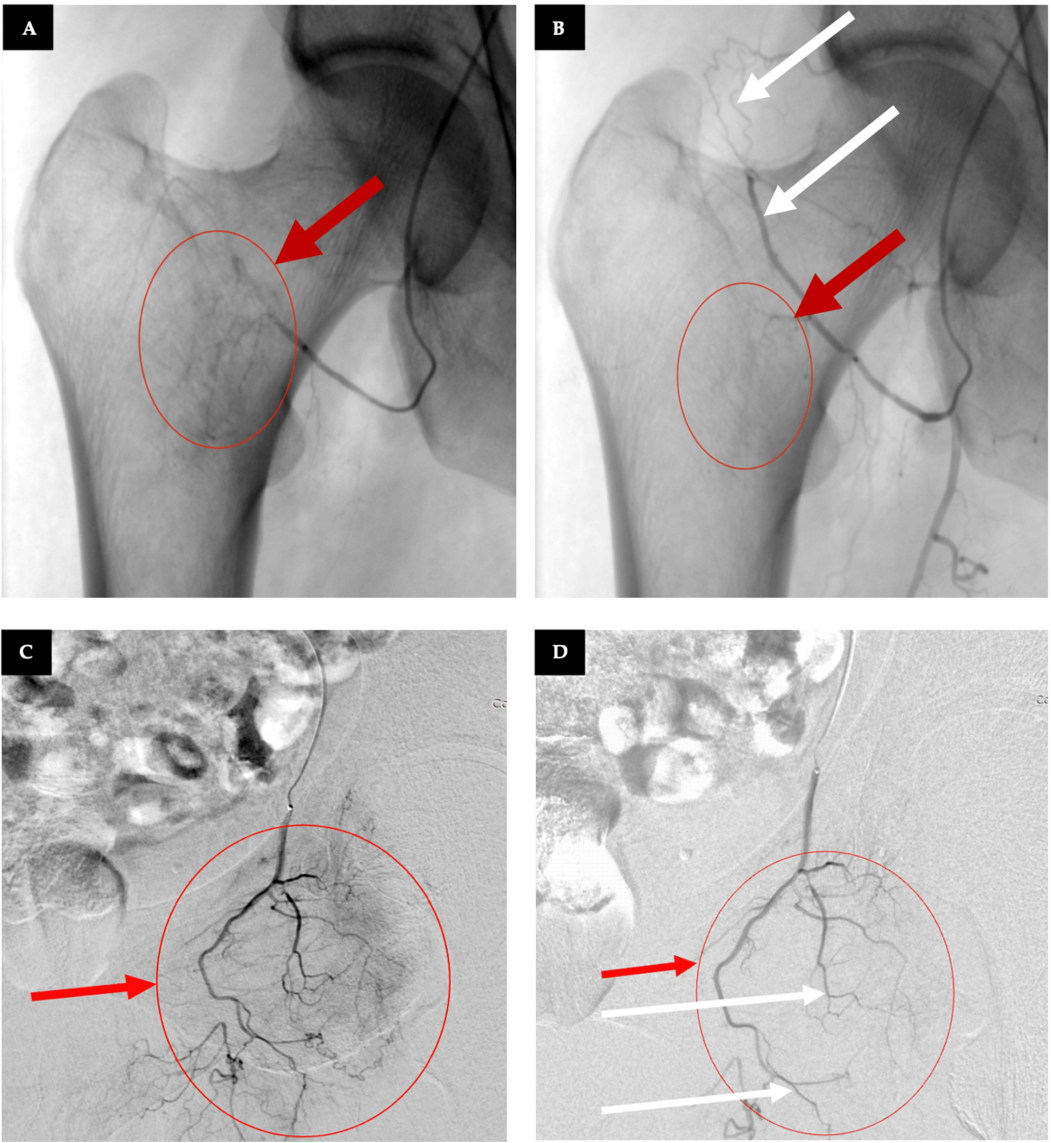
Fluoroscopic views of the angiogenesis zones respective to the lateral circumflex femoral artery (A, B) and obturatory artery (C, D). (A) Lateral circumflex femoral artery pre-embolization blush - red circle and arrow and (B) post-embolization. (C) Obturatory artery pre-embolization blush - red circle and arrow and (D) post-embolization. White arrows (B, D) indicate sustained distal perfusion.

The angiographic machine is positioned in an ipsilateral oblique and craniocaudal view to differentiate the inferior gluteal artery (IGA) and the superior gluteal artery. The MP is advanced to the origin of the IGA, where a contrast injection is administered, delineating the pudendal and obturator arteries (OA). A 0.021-inch microcatheter (MC) (Direxion, Boston Scientific, Marlborough, MA, USA) is then introduced to selectively cannulate the distal OA and distal IGA. Following this, the MP is retracted to the common IA and advanced into the external IA, checking for any additional vessels supplying the femoral head before proceeding into the common femoral artery, reaching the origin of the profunda femoris artery at the level of the inguinal ligament. At this point, contrast injection via the MP is performed to visualize the lateral circumflex femoral artery (LCFA). At this point, the MC is utilized for supraselective TAE of the LCFA. Fluoroscopic imaging (blank roadmap) is used to monitor the resolution of distal vascularity, ensuring that TAE is effective while maintaining sufficient blood flow within the targeted artery branch, as illustrated in Figure 3. The procedure concludes with a control angiogram, followed by achieving hemostasis through manual compression of the brachial artery. We report the following details: embolic suspension was continuously agitated during delivery to avoid sedimentation; injection proceeded under real-time fluoroscopy in 0.2-0.3 mL aliquots until near stasis was achieved, with avoidance of reflux into non-target branches; and radiation exposure (dose-area product) and contrast volume were documented for each patient. All procedures were performed by the same interventional radiologist with over five years of experience in musculoskeletal TAE (FB). Patients were observed for at least four hours post-procedure with monitoring of vital signs, puncture site, and neurovascular status before and subsequently discharged after the intervention.

Statistical analysis

Descriptive statistics (mean ± standard deviation) were calculated for the VAS and HHS at baseline, one, four, eight, and twelve weeks. Normality of the paired differences was assessed using the Shapiro-Wilk test. Adjacent time point comparisons (1 week-0 weeks, 4 weeks-1 week, 8 weeks-4 weeks, and 12 weeks-8 weeks) were evaluated using paired-samples t-tests. To control for multiple hypothesis testing, p-values were adjusted using the Holm procedure across the four planned comparisons for each outcome. A two-sided p-value <0.05 was considered statistically significant. Statistical analyses were performed using GraphPad Prism 9 (v9.4.1) and Python.

Ethics statement

The study protocol received approval from the Institutional Review Board of Monza Oncology Hospital (approval number: 1134/2022) and was conducted in accordance with the Declaration of Helsinki and compliant with established ethical standards and regulatory guidelines.

## Results

Table 2 summarizes the baseline demographic and clinical profile of the cohort. No significant associations were observed between patient characteristics and postprocedural outcomes. All patients completed the scheduled follow-up visits with a total observation period of 12 weeks (range = 83 to 94 days from the procedure date). None of the procedures were associated with technical failures or intraprocedural complications. The mean fluoroscopy time was 22.1 ± 11.4 minutes. The corresponding estimated dose-area product was approximately 210 Gy·cm².

**Table 2:** Patient demographics and characteristics. SD = standard deviation; BMI = body mass index; OA = osteoarthritis; NSAIDs = nonsteroidal anti-inflammatory drugs

Characteristic	
Age (years), mean ± SD	67.2 ± 5.7
Height (cm), mean ± SD	165 ± 8.1
Weight (kg), mean ± SD	89.3 ± 13.5
BMI, mean ± SD	32.8 ± 4.9
Gender, male (%)/female (%)	7 (39%)/11 (61%)
Smokers, n (%)	3 (17%)
Bilateral hip OA, n (%)	6 (33%)
Knee OA, n (%)	3 (17%)
Lumbar spine OA, n (%)	2 (11%)
Hand/Wrist OA, n (%)	1 (6%)
Daily physical activity, n (%)	4 (22%)
Previous opioid usage, n (%)	7 (39%)
Previous NSAIDs usage, n (%)	17 (94%)
Physiotherapy, n (%)	9 (50%)
Intra-articular injections, n (%)	6 (33%)

Several patients presented with more than one comorbidity, particularly cardiovascular disease and obesity. Bilateral hip symptoms were reported in six patients, with the more symptomatic side consistently selected for TAE. Three patients also had concomitant knee osteoarthritis, two had lumbar spine involvement, and one had hand osteoarthritis.

Harris Hip Score

The HHS improved by 14.4 points at one week (from 45.5 ± 4.7 to 59.9 ± 7.1, p < 0.01), followed by a smaller gain of 0.9 points at week four (60.8 ± 8.0), and concluded with a total increase of 17.1 points from baseline at week eight (62.6 ± 6.0). At 12 weeks, the score remained stable. While the change from baseline remained statistically significant at all time points, no statistically significant differences were observed for the consecutive follow-ups after week one (Table 3). These findings suggest that functional recovery and pain relief occurred early and remained stable.

**Table 3:** Harris Hip Score evolution across time points postprocedure. *: Adjacent time-point comparisons were analyzed using paired t-tests (two-sided) with Holm correction across four comparisons; t-values represent the magnitude of change relative to the previous time point. SD = standard deviation

Harris Hip Score	Mean ± SD	*P-value (vs. previous follow-up)	t-value
Baseline	45.5 ± 4.68	NA	NA
One week	59.9 ± 7.10	0.000004	7.42
Four weeks	60.8 ± 8.02	0.48	0.71
Eight weeks*	62.6 ± 6.01	0.41	1.31
Twelve weeks*	62.1 ± 7.45	0.277430	1.78

**Figure 4:**
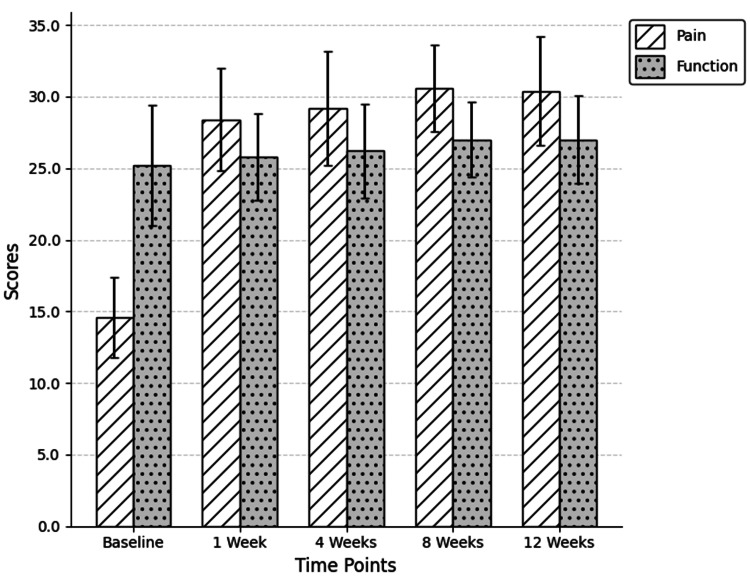
Evolution of HHS pain function subscale scores following TAE. Mean and standard deviation are represented at each follow-up time. HHS = Harris Hip Score; TAE = transarterial embolization

HHS pain subscale scores doubled from baseline (14.6 ± 2.8) to one week (28.4 ± 3.6) and peaked at eight weeks (30.6 ± 3.0), reflecting marked analgesic benefit (Figure 4). Gait and activities subdomain scores showed modest but consistent gains over time, while the deformity subscore remained largely stable. Functional scores (range of motion) remained unchanged until eight weeks but dropped at 12 weeks (2.5 ± 0.5), reflecting subjective or mechanical limitations.

Visual Analog Scale

The mean VAS score decreased by 3.6 points at one week (from 7.8 ± 1.3 to 4.2 ± 2.0, p < 0.01), remained stable at four weeks (4.2 ± 2.4), improved by 0.5 points by week eight (3.7 ± 2.7), and concluded with a reduction of 3.5 points from baseline at 12 weeks (4.3 ± 2.2). These values indicate a significant and sustained reduction in pain following the intervention (Table 4).

**Table 4:** Visual Analog Scale score evolution across time points postprocedure. *: Pairwise adjacent time-point comparisons were performed using paired-samples t-tests (two-sided) with Holm correction for four multiple comparisons; t-values represent the magnitude of change relative to the previous time point. SD = standard deviation

Visual Analog Scale	Mean ± SD	*P-value	t-value
Baseline	7.8 ± 1.3	NA	NA
One week	4.2 ± 2.0	0.00002	6.69
Four weeks	4.2 ± 2.4	0.93	0.09
Eight weeks	3.7 ± 2.7	0.75	0.91
Twelve weeks	4.3 ± 2.2	0.78	1.16

**Figure 5:**
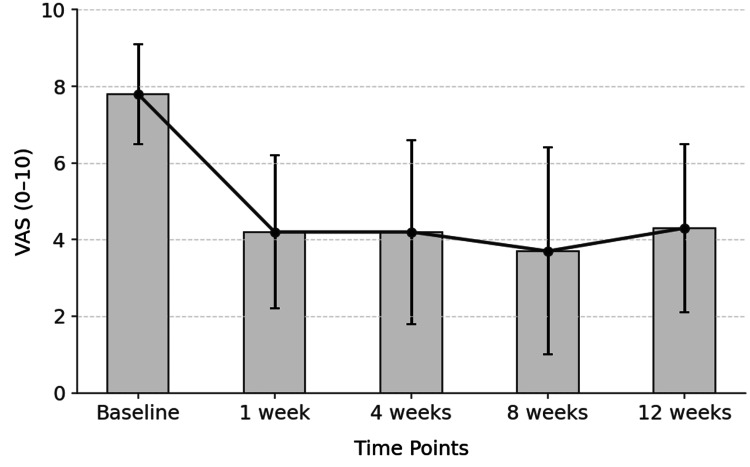
Evolution of mean VAS scores over time points. Mean and standard deviation are represented at each follow-up time. VAS = Visual Analog Scale

No statistically significant differences were observed between consecutive follow-ups beyond week one. The majority of the analgesic benefit occurred within the first week, followed by a stable plateau phase without further significant pain reduction (Figure 5).

## Discussion

In this prospective study, hip TAE in elderly patients with Tönnis grade I-II HOA led to early and sustained improvements in pain with no major periprocedural complications. Both patient-reported and clinician-assessed outcomes (VAS and HHS) demonstrated clinically meaningful gains that were maintained throughout the 12-week follow-up. The parallel rise in patient-reported and clinician-assessed scores strengthens the evidence that the intervention is effective and technically feasible. Evidence for hip TAE remains limited. The earliest description of TAE for inflammatory hip synovitis documented qualitative pain relief and better walking ability but used no formal outcome score [17]. Correa et al. later treated 13 patients, used the Western Ontario and McMaster Universities Osteoarthritis Index for assessment, and observed significant improvement at six months [15]. Our inclusion of VAS and clinician-assessed outcomes helps fill an important measurement gap, and consistent with those earlier studies, we observed no requirement for immediate conversion to THA and no immediate acceleration of symptoms after the procedure.

As the hip data state of the art is limited, it is important to compare the larger experience with genicular artery embolization (GAE) for knee osteoarthritis. Prospective studies and randomized controlled trials consistently demonstrate VAS reductions of 3.0-4.1 points and parallel patient-reported outcome measure gains at 12 months [4,5], findings mirrored in our own knee osteoarthritis cohort, where significant improvements persisted from one to twelve months without complications [8]. Several groups have followed knee GAE patients for up to 24 months and documented durable pain relief [18]. These longer-term knee osteoarthritis GAE data show that the procedure has prolonged clinical benefit in a weight-bearing joint and provide a useful benchmark for the hip. This exploratory pilot study includes a small cohort (n = 18) and no control group, which limits interpretation. The 12-week follow-up captures early clinical response but does not yet inform on durability, which remains to be evaluated in studies with longer surveillance periods. Although the consistent findings across both VAS and HHS suggest a treatment effect, we cannot exclude placebo responses or regression to the mean in actively symptomatic patients undergoing a novel intervention.

A persistent concern is whether occluding the branches of the LCFA might precipitate osteonecrosis or other ischemic complications [8]. In earlier hip reports and in the knee literature, no clinical or MRI evidence of osteonecrosis was detected during the first year of follow-up [18]. Previously reported minor events, such as transient posterior thigh numbness, probably related to non-target TAE of small sciatic nerve branches, have been rare [15].

Equally unsettled is the durability of benefit: published hip studies extend beyond six to twelve months, so the long-term relief documented after knee GAE [18] cannot yet be assumed for the hip. Careful angiographic mapping, calibrated microspheres or resorbable embolics, and routine postprocedural imaging surveillance remain essential precautions until larger data sets become available.

Regarding safety, TAE has a favorable profile in hip, knee, and other applications. The most frequently reported short-term events include minor inguinal hematomas, transient skin discoloration, temporary pain flares, and self-limited paresthesia [4,19]. These effects are mild, typically resolve within days to weeks, and rarely require intervention. A recent meta-analysis of over 300 knees reported transient skin changes in 11.6% of cases, all of which resolved without ulceration; rare acute events such as vasculitis or deep vein thrombosis were anecdotal [19]. No evidence of permanent neurologic deficits or tissue necrosis has been reported [4,20]. Longer-term data, while currently limited, remain reassuring. Prospective follow-ups up to 12 months show sustained improvements in pain and function without emergence of osteonecrosis or structural bone compromise [21]. Across studies with up to 24 months of follow-up, no cases of osteonecrosis or progressive structural joint damage have been observed on imaging, and late-onset complications such as delayed ischemia, chronic neuropathy, or ulceration have not been reported [21]. Patient-reported outcomes confirm lasting benefit without chronic adverse experiences. Recurrence of symptoms in some patients appears related to osteoarthritis progression rather than procedure-related harm [9]. Cautious patient selection is essential, and adverse events of TAE appear confined to transient, minor, self-limiting effects with no signal of long-term joint compromise.

If future work confirms that HOA TAE delivers meaningful 12-month pain and functional gains in elderly patients who are unsuitable or unwilling for THA, the procedure can gain an intermediate position in current treatment algorithms and guidelines, which now progress from conservative measures (physiotherapy, oral or topical analgesics, intra-articular injections) directly to surgery [9,10]. Earlier pain control might also reduce prolonged opioid requirements and the systemic adverse effects associated with long-term analgesic use [22,23]. Other minimally invasive treatment options for osteoarthritis-related joint pain include intra-articular corticosteroid or hyaluronic acid injections, platelet-rich plasma, radiofrequency ablation of sensory nerves, and percutaneous nerve blocks [23,24]. Subchondroplasty and MRI-guided focused ultrasound have also been used in selected cases [25]. Comparatively, TAE targets the abnormal neovascularization and accompanying inflammatory cascade within periarticular soft tissues. Studies comparing TAE with radiofrequency ablation and injectable therapies suggest that while radiofrequency ablation procedures achieve the largest mean pain score reduction at one year, consistent with our findings, TAE produces consistent improvements across multiple timepoints with comparable functional gains [26]. TAE offers specific advantages: sustained pain relief over several months to years [8,27]; a low complication rate limited to transient discomfort or numbness [25-27]; and feasibility in patients with contraindications to surgery or repeated intra-articular injections [28]. Multiple studies report significant reductions in analgesic use following TAE [25,29].

The brachial approach permits immediate ambulation and same-day discharge, while also enabling the physician to perform the procedure bilaterally [28]. The present literature, including our own study, is limited by small sample size, modest observation windows, and the absence of control groups. As this was an exploratory pilot study, the cohort was intentionally restricted by strict eligibility criteria and institutional feasibility. The lack of a parallel control group limits the ability to attribute observed improvements only to the TAE procedure. Although the consistent and clinically meaningful gains across multiple outcome measures suggest a treatment effect, placebo responses and regression to the mean cannot be fully excluded. There is a potential for selection bias due to single-center recruitment and eligibility criteria. Participants and assessors were not blinded, so expectation effects cannot be excluded. Future studies should incorporate randomized controlled designs or at least matched conservative treatment comparators to better delineate the true efficacy of hip TAE.

Given the procedural novelty, lack of published prospective data, and ethical constraints against placebo TAE, in this subgroup, we adopted a single-arm format as an initial step. Some patients presented with bilateral hip symptoms or concomitant osteoarthritis at other sites, which may have contributed to functional limitations. While prior conservative therapies were common in our cohort, no new interventions were introduced during follow-up.

Comparable early experiences with knee TAE have shown pain and functional improvements using bioresorbable microspheres in high-risk patients [28]. Although anatomical and biomechanical differences exist between the hip and knee, such findings provide external support for the feasibility of TAE in load-bearing joints and reinforce the rationale for expanding prospective, controlled trials in HOA [29].

As several participants presented with more than one comorbidity, including cardiovascular and pulmonary conditions, some of the limitations in physical activity and functional scores can be attributable to these systemic diseases rather than HOA alone. This overlap should be considered when interpreting functional outcome improvements.

The biological pathways by which selective TAE of hypervascular periarticular tissue produces sustained analgesia remain incompletely understood [4]. Future research should extend radiological and clinical surveillance beyond two years, integrate advanced imaging or biomarker analyses to clarify the mechanism, and undertake adequate trials that compare TAE with intra-articular injections, radiofrequency ablation, or delayed arthroplasty.

## Conclusions

This pilot study suggests that TAE of the hip provides rapid pain relief and early functional gains in elderly patients with Tönnis I-II osteoarthritis who are not candidates for arthroplasty. Most improvement occurred within the first week and was maintained through 12 weeks. The procedure was technically feasible via brachial access and was not associated with major complications in this cohort. These findings support hip TAE as a minimally invasive bridge between conservative therapy and surgery for high-risk or surgery-averse patients. Larger comparative studies with longer observation are needed to confirm durability, refine patient selection, and define the role of TAE in care pathways.
